# Transcriptional signature of prion-induced neurotoxicity in a *Drosophila* model of transmissible mammalian prion disease

**DOI:** 10.1042/BCJ20190872

**Published:** 2020-02-28

**Authors:** Alana M. Thackray, Brian Lam, Anisa Shahira Binti Ab Razak, Giles Yeo, Raymond Bujdoso

**Affiliations:** 1 Department of Veterinary Medicine, University of Cambridge, Madingley Road, Cambridge CB3 0ES, U.K.; 2 Metabolic Research Laboratories and MRC Metabolic Diseases Unit, Wellcome Trust-MRC Institute of Metabolic Science, Addenbrooke's Hospital, Cambridge CB2 0QQ, U.K.

**Keywords:** *Drosophila melanogaster*, neurodegeneration, prion, transcriptomics

## Abstract

Prion diseases are fatal transmissible neurodegenerative conditions of humans and animals that arise through neurotoxicity induced by PrP misfolding. The cellular and molecular mechanisms of prion-induced neurotoxicity remain undefined. Understanding these processes will underpin therapeutic and control strategies for human and animal prion diseases, respectively. Prion diseases are difficult to study in their natural hosts and require the use of tractable animal models. Here we used RNA-Seq-based transcriptome analysis of prion-exposed *Drosophila* to probe the mechanism of prion-induced neurotoxicity. Adult *Drosophila* transgenic for pan neuronal expression of ovine PrP targeted to the plasma membrane exhibit a neurotoxic phenotype evidenced by decreased locomotor activity after exposure to ovine prions at the larval stage. Pathway analysis and quantitative PCR of genes differentially expressed in prion-infected *Drosophila* revealed up-regulation of cell cycle activity and DNA damage response, followed by down-regulation of eIF2 and mTOR signalling. Mitochondrial dysfunction was identified as the principal toxicity pathway in prion-exposed PrP transgenic *Drosophila*. The transcriptomic changes we observed were specific to PrP targeted to the plasma membrane since these prion-induced gene expression changes were not evident in similarly treated *Drosophila* transgenic for cytosolic pan neuronal PrP expression, or in non-transgenic control flies. Collectively, our data indicate that aberrant cell cycle activity, repression of protein synthesis and altered mitochondrial function are key events involved in prion-induced neurotoxicity, and correlate with those identified in mammalian hosts undergoing prion disease. These studies highlight the use of PrP transgenic *Drosophila* as a genetically well-defined tractable host to study mammalian prion biology.

## Introduction

Protein misfolding neurodegenerative diseases are invariably fatal conditions that include Alzheimer's disease, Huntington's disease, Parkinson's disease, motor neuron disease, tauopathies and prion diseases [1,2]. These conditions are caused by the accumulation of disease-specific misfolded protein in the brain of affected individuals [3]. Prion diseases, which include scrapie of sheep, bovine spongiform encephalopathy (BSE) of cattle, together with Creutzfeldt–Jakob disease (CJD) in humans [4], are the prototypic protein misfolding neurodegenerative disorders. Furthermore, prion diseases are unique since they are naturally transmissible between individuals of the same and different species. In this context prion diseases are an important paradigm for protein misfolding neurodegenerative conditions in general since Alzheimer's, Huntington's, Parkinson's and motor neuron disease, as well as tauopathies, show prion-like transmission under experimental settings, evidenced by the transcellular spread of misfolded disease-specific cytosolic protein [5].

Although advances have been made in providing an understanding of the mechanism of prion-induced neurotoxicity this process remains to be fully defined. Conversion of the normal host protein PrP^C^, a plasma-membrane bound GPI-anchored protein, into the abnormal form PrP^Sc^, the transmissible prion agent, is central to prion disease neurotoxicity [4,6]. This is shown by the failure of PrP^Sc^ to cause pathology in brain tissue that lacks PrP^C^ expression [7,8] and by the reversal of neurodegeneration when PrP^C^ expression is down-regulated during prion disease [9–11]. In this context, prion-induced neurotoxicity may arise from one or more of the following mechanisms: loss of PrP^C^ function, toxic gain of function by PrP^Sc^, generation of a toxic intermediate or by-product during PrP conversion [12,13]. Alternatively, interference with the normal biosynthesis and metabolism of PrP^C^ mediated by the presence of PrP^Sc^ may play a role in prion-induced toxicity [14]. For example, PrP can accumulate in the cytosol in a misfolded form when proteasomal activity is compromised [15,16] and cytosolic PrP has been reported to be neurotoxic in certain neurons [17–20]. It has been shown that the accumulation of PrP^Sc^ in cells [21] and mice [22] triggers over-activation of the unfolded protein response with a resultant block of protein translation. Despite these advances in understanding the mechanism of prion-induced neurotoxicity this process remains to be fully defined. To do so requires identification of cellular events that lead to, and accompany, inhibition of protein synthesis in prion-infected hosts.

Prion diseases are difficult to study in their natural hosts, such as humans and ruminants because these diseases can take many years to develop, resulting in progress being slow and cumbersome [4]. In addition, the natural forms of prion diseases tend to occur in outbred populations, which renders genetic analysis of complex biochemical pathways difficult. Even in the more amenable experimental system of mouse models, the significant expense of both time and husbandry restrict the scope of genetic experimentation for dissection of prion disease mechanisms. In order to circumvent these issues, we have established *Drosophila* as a new tractable animal model of transmissible mammalian prion disease. To do so, we generated *Drosophila* transgenic for ovine PrP with an N-terminal leader peptide and C-terminal GPI anchor sequence that is targeted to the plasma membrane [PrP(GPI)] [23]. Adult *Drosophila* transgenic for pan neuronal expression of ovine PrP(GPI) exposed to scrapie prions, at the larval stage, authentically replicate mammalian prions and develop a neurotoxic phenotype evidenced by a decrease in locomotor ability [24]. This prion-induced neurotoxic fly phenotype showed hallmark features of bona fide mammalian prion disease, namely accumulation of Proteinase K (PK)-resistant PrP^Sc^, prion seeding activity and the propagation of prions that are transmissible to mice. In addition, we generated *Drosophila* transgenic for ovine PrP that lack an N-terminal leader peptide and C-terminal GPI anchor sequence, which is targeted to the cytosol [PrP(Cyt)]. Adult PrP(Cyt) *Drosophila* show a fly-to-fly transmissible toxicity after exposure to exogenous ovine prions at the larval stage [25]. Collectively, these PrP transgenic *Drosophila* provide novel tractable experimental hosts for the study of mammalian prion biology.

Here we have performed an RNA-Seq-based transcriptome analysis of prion-infected *Drosophila* in order to search for canonical and toxicity pathways involved in prion-induced neurotoxicity. Our analysis has revealed that during the early phase of prion infection in PrP(GPI) transgenic *Drosophila*, there was up-regulation of genes associated with cell cycle activity and DNA damage repair, followed by down-regulation of the protein synthesis regulation pathways eIF2 and mTOR (mechanistic target of rapamycin) signalling. In addition, mitochondrial dysfunction was identified as the principal toxicity pathway in prion-exposed *Drosophila*. These data indicate that aberrant cell cycle activity, repression of protein synthesis and altered mitochondrial function are key events involved in prion-induced neurotoxicity. Our studies show that PrP transgenic *Drosophila,* a genetically well-defined tractable host, represent a new model for the study of prion-induced neurotoxicity, an important aspect of transmissible mammalian prion biology.

## Materials and methods

### Drosophila fly lines

The *UAS*-PrP(GPI) fly line w; M{VRQ-PrP(GPI), 3xP3-RFP.attP}ZH-51D transgenic for ovine V^136^R^154^Q^171^ (VRQ) PrP expressed with an N-terminal leader peptide and C-terminal signal sequence [PrP(GPI)] was generated by PhiC31 site-specific transformation as previously described [23]. The *UAS*-PrP(Cyt) fly line w; M{VRQ-PrP(Cyt), 3xP3-RFP.attP}ZH-51D transgenic for ovine VRQ that lacked an N-terminal leader peptide and C-terminal signal sequence [PrP(Cyt)] was generated by PhiC31 site-specific transformation as previously described [25]. *Cre*-mediated removal of RFP from the fly genome of both VRQ PrP variants and from non-transgenic control 51D *Drosophila* was performed by conventional fly crosses [26]. The *Elav-GAL4* (P{w[ + mW.hs] = GawB}elav[C155]) was obtained from the Department of Genetics, University of Cambridge. All fly lines were raised on standard cornmeal media [27] at 25°C, maintained at low to medium density. Flies were used in the assays described below or harvested at various time points and then frozen at −80°C until required.

### Prion inoculation of *Drosophila*

*Drosophila* at the larval stage of development were exposed to brain homogenates of cerebral cortex tissue from confirmed scrapie-positive or known scrapie-negative sheep. The scrapie-infected isolate was prepared from a terminal scrapie-affected sheep identified by routine statutory surveillance (VRQ/VRQ isolate SE1848/0005) [28] that showed typical vacuolar pathology in the medulla oblongata of the brain stem and that was positive for disease-associated PrP as judged by immunohistochemistry or western blot. New Zealand-derived VRQ/VRQ scrapie-free brain tissue was used as control material. Two hundred and fifty microlitres of a 1% brain homogenate prepared in PBS pH7.4 were added to the top of the cornmeal that contained third instar *Drosophila* larvae in 3″ plastic vials. Flies were transferred to fresh vials, that lacked any inoculum, following eclosion.

### Locomotor assay

The locomotor ability of flies was assessed in a negative geotaxis climbing assay as described previously [29]. Briefly, age-matched, pre-mated female flies (3 × *n* = 15 for each genotype at the start of the experiment) were placed in adapted plastic 25 ml pipettes that were used as vertical climbing columns. The flies were allowed to acclimatise for 30 min prior to the assessment of their locomotor ability. Flies were tapped to the bottom of the pipette (using the same number and intensity of taps) and then allowed to climb for 45 s. At the end of the climbing period the number of flies above the 25 ml mark, the number below the 2 ml mark and the number in between the 2 ml and 25 ml mark were recorded. This procedure was performed three times at each time point. The mean ± SD performance index (PI) for each group of flies was calculated as described [29]. Statistical analysis of the data was performed using one-way analysis of variance (ANOVA), together with Tukey honestly significant difference (HSD) for *post hoc* analysis or the unpaired samples *t*-test using Prism (GraphPad Software Inc, San Diego, U.S.A.).

### Preparation of fly head homogenates and RNA extraction

*Drosophila* were exposed at the larval stage to either scrapie-infected or prion-free sheep brain homogenate [24,26]. After hatching, *Drosophila* were transferred to inoculum-free tubes and allowed to develop normally. At 5 days and 40 days post hatching, groups of female flies (three sets of *n* = 15, i.e. 45 flies in total per treatment group) were euthanized and then decapitated. This was achieved by placing whole flies in eppendorf tubes, which were then frozen in liquid nitrogen for 10 min and then vortexed for 2 min. Individual fly heads were then isolated and placed in clean eppendorf tubes, using a paint brush, for head homogenate preparation. All subsequent procedures for RNA preparation were performed at 18°C unless otherwise stated. Each set of 15 fly heads was homogenised by manual grinding in an eppendorf tube with 50 µl Trizol (Cat No. 15596-018; Invitrogen). A further 50 µl of Trizol were mixed with the homogenate, which was then microfuged at 16,160×*g* for 10 min and the supernatant transferred to RNAse-free tubes before adding a further 50 µl Trizol and incubated for 5 min. RNA was isolated by conventional chloroform:isoamylalcohol:isopropanol extraction followed by ethanol precipitation. Air-dried RNA pellets were treated with DNAse 1 (Cat No. EN0521; Thermo Scientific) at 37°C for 60 min, with the reaction halted by the addition of EDTA and incubation at 65°C for 10 min. RNA samples were then subjected to the Qiagen RNeasy RNA Cleanup protocol according to the manufacturers’ instructions. The RNA samples were eventually eluted from RNeasy mini-spin columns, ethanol precipitated, re-suspended in RNAse-free water and stored at −80°C.

### Transcriptome mRNA sequencing

Transcriptome profiling was assessed using RNA sequencing performed at the High-performance computing cluster at the Research Computing Service, University of Cambridge. A library of mRNA was prepared from 200 ng of total RNA, extracted as described above and that had been originally pooled from 15 fly heads from each experimental condition, using the Illumina TruSeq Stranded mRNA Library Prep kit (Illumina, San Diego, CA, U.S.A.) according to the manufacturer's protocol. Briefly, poly-A containing mRNA was purified from total RNA using poly-T oligo attached magnetic beads and fragmented using divalent cations and elevated temperature. Cleaved mRNA was copied into first strand cDNA using Superscript II reverse transcriptase and random primers in the presence of Actinomycin D. Strand specificity was achieved by replacing dTTP with dUTP in the second strand marking mix, followed by second strand cDNA synthesis using DNA Polymerase I and RNase H. Blunt ended double stranded cDNA was adenylated at the 3′ end to allow ligation of T-linked single-index adapters to the fragment to generate the cDNA library. Products were enriched with 15 cycles of PCR and purified with AMPure XP magnetic beads to create the final cDNA library. Libraries from individual samples were combined at equal molar concentration of DNA, before loading onto one lane of either an Illumina HiSeq 2000 instrument [in the case of PrP(GPI)] or 3 lanes of a HiSeq 4000 instrument [in the case of PrP(Cyt) and 51D] for single-end sequencing of 40 bp [in the case of PrP(GPI)] and 50 bp [in the case of PrP(Cyt) and 51D] products. Sequencing was performed at the Genomics Core, Cancer Research UK Cambridge Institute, Cambridge.

### Bioinformatics analysis

The quality of sequence reads was examined using FastQC while Multiple Genome alignment (MGA) was used to rule out contamination from other DNA sources throughout the experimental procedures. A total of 945.7 million reads, or 17.5 ± 1.3 million reads per sample, was acquired for the study presented here. A total of 83.4% of sequence reads were successfully mapped onto the *Drosophila melanogaster* reference genome version BDGP6 released from the Berkeley *Drosophila* Genome Project [30] using TopHat version 2.0.11 [31]. Raw gene-level abundance was determined through the use of Htseq-count (version 0.6.1p1). Trimmed mean of M values normalisation and differential gene expression analysis was performed using EdgeR and Limma. The normalised gene abundance is represented in log2CPM, which stands for the base-2 logarithm of Count per Million. The raw sequencing data and the comparison tables are available on-line at Gene Expression Omnibus (accession number GSE144028). Gene annotations and pathway analysis were performed using Ingenuity Pathway Analysis (IPA) (Qiagen, U.S.A.). These analyses identified canonical pathways or toxicity functions from the IPA library that were most relevant to the input *Drosophila* data set. The relevance of the association between the input data set and the identified pathway or toxicity function was quantified by two methods. Firstly, Fisher's exact test was used to calculate a *P*-value that determined whether the probability of association between the genes in the *Drosophila* data set and the proposed pathway could be attributed to chance alone. Secondly, the ratio of the number of genes from the *Drosophila* data set that map to the pathway divided by the total number of molecules that map to the pathway or toxicity function was calculated. The statistical computing package P- heatmap was used for visualisation of differentially expressed genes in each genotype of prion-exposed *Drosophila*. The changes visualised for each specific gene in the heat map presentations refer to the log2-fold change between expression in scrapie-exposed versus prion-free sheep brain homogenate-exposed *Drosophila*.

### Quantitative PCR (qPCR)

*Generation of cDNA:* One microgram of target RNA was used per reverse transcription (RT) reaction to generate cDNA with 20 Units of Superscript II reverse transcriptase (catalogue no. 18064-022; Gibco Life Technologies) at 42°C for 60 min in the presence of 50 mM Tris–HCl [pH8.3], 75 mM KCl, 3 mM MgCl_2_, 5 mM dithiothreitol, 0.5 mM deoxynucleoside triphosphates, 1 Unit of RNase H (catalogue no. M428; Promega), and 0.1 µg of oligo(dT)_15_ (catalogue no. C1101; Promega). For every reaction set, one RNA sample was performed without Superscript II reverse transcriptase to provide a negative control in subsequent PCRs. *Primers:* PCR primers were used for the following *Drosophila* genes: cdc2; dPCNA; Cyclin A; Cyclin B; eIF2α, eIF4A; eIF4E; eIF3 S10 (see Supplementary Data S1 for sequences). To compensate for variations in amounts of input RNA and efficiency of the reverse transcription, an endogenous housekeeping gene actin was also quantified, and results were normalised to these values. *qPCR amplification:* qPCR was performed in an ABI 7900HT real-time PCR system using a 384 well plate format with individual amplification reaction volumes of 15 µl that contained 2.5 µl of cDNA sample (5.5 ng cDNA per well), 1 µl forward primer and 1 µl of reverse primer (both at 10 µM) (see Supplementary Data for primer sequences), 7.5 µl of Sybr Green PCR Mastermix (Cat No. 4309155; Applied Biosystems) and 3 µl sterile, RNA free water. Each qPCR amplification was performed in triplicate wells with the following conditions: 2 min at 50°C and 10 min at 95°C, followed by a total of 45 two-temperature cycles (15 s at 95°C and 1 min at 60°C). Quantification of signal was achieved by setting thresholds within the logarithmic phase of the PCR for the target gene and the housekeeping gene actin, and determining the cycle number at which the threshold was reached (Ct) for both. The relative amount of the target gene transcript expression was plotted as mean 2^ΔCt^ ± standard error of the mean for each treatment group, where ΔCt = (Ct for the target gene sample) − (Ct for the equivalent actin sample). Statistical analysis was performed by multiple group analysis of 2^ΔCt^ values within each treatment group using one-way analysis of variance (ANOVA), together with Tukey HSD for *post hoc* analysis.

## Results

### *Drosophila* model of transmissible mammalian prion disease

We have previously established that adult PrP transgenic *Drosophila* show a neurotoxic phenotype after exposure to mammalian prions at the larval stage [26]. Supplementary Data S2 show that the locomotor activity of prion-exposed PrP(GPI) *Drosophila* was significantly decreased compared with the response by similar flies exposed to control scrapie-free sheep brain homogenate (Supplementary Data S2A). The prion-induced neurotoxic fly phenotype was biologically relevant since it was associated with hallmark features of bona fide mammalian prion disease, namely accumulation of PK-resistant PrP^Sc^, prion seeding activity and the propagation of prions that are transmissible to mice [24]. *Drosophila* transgenic for PrP(Cyt), also showed a locomotor defect that was more pronounced than PrP(GPI) *Drosophila* (Supplementary Data S2B). In contrast with these data, adult control non-transgenic 51D *Drosophila* showed no difference in locomotor activity after exposure to scrapie-infected or healthy prion-free sheep brain material at the larval stage (Supplementary Data S2C). Here we have used this invertebrate model of transmissible mammalian prion disease to perform RNA-Seq-based transcriptome analysis of scrapie- and mock-infected *Drosophila* in order to determine the cellular and biochemical pathways affected in the fly as a consequence of prion-induced neurotoxicity.

### Temporal and quantitative gene expression changes in prion-exposed PrP *Drosophila*

RNA-Seq-based transcriptome analysis was performed on adult fly head homogenate prepared from *Drosophila* exposed at the larval stage to either scrapie-infected or prion-free sheep brain homogenate [24,26]. Each *Drosophila* treatment group was represented by triplicate samples of 15 fly heads. A total of 9672 *Drosophila* genes were collectively detected in prion-infected and control fly samples harvested at 5 days and 40 days post hatching. This represented expression of >61.7% of the *Drosophila melanogaster* genome during the course of the experiment.

**Figure 1.**
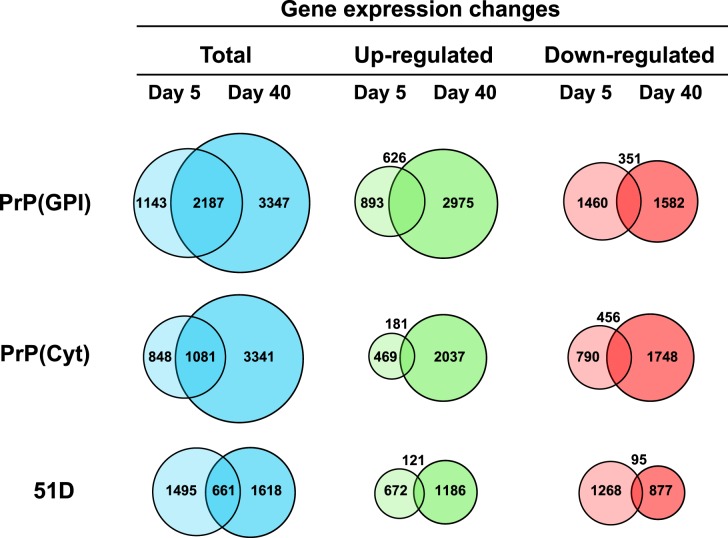
Temporal alterations in *Drosophila* gene expression following prion infection. Venn diagram representation of the number of prion-specific differentially expressed genes in *Drosophila* at 5 and 40 days post hatching following exposure to scrapie-infected sheep brain homogenate at the larval stage.

We determined the number of genes differentially expressed in each *Drosophila* treatment group at 5 days and 40 days post hatching. Differentially expressed genes were identified as those with ≥2-fold change in expression level (either increased or decreased between scrapie-infected and mock-infected flies) with a false discovery rate of 5%. The data in Figure 1 show that the total number of genes differentially expressed in *Drosophila* as a consequence of prion infection was of the order PrP(GPI) > PrP(Cyt) > 51D. All three genotypes of *Drosophila* showed a similar number of differentially expressed genes at 5 days post hatching. However, more genes were differentially expressed in adult prion-exposed PrP *Drosophila* aged 40 days compared with those at 5 days of age, which was due principally to a greater number of up-regulated genes. The number of down-regulated genes was similar at 5 days and 40 days of age in prion-exposed adult PrP(GPI) *Drosophila* but elevated at day 40 in PrP(Cyt) flies. The fact that control 51D *Drosophila* did not show any significant temporal change in the total number of differentially expressed genes, or their regulation profile, implies that the gene changes seen in PrP(GPI) and PrP(Cyt) *Drosophila* were prion-induced and not simply an effect of these flies ageing.

### EIF2 signalling and cell cycle activity are perturbed in prion-exposed PrP(GPI) *Drosophila*

We next performed pathway enrichment analysis using combined lists of differentially expressed genes in prion-exposed PrP *Drosophila* on both day 5 and 40 in order to obtain unbiased predictions of cellular and biochemical events perturbed (involved) in each fly line as shown in Table 1. The data in Table 1A show the top 5 canonical pathways perturbed in PrP *Drosophila* as a consequence of prion-exposure. The most statistically relevant canonical pathway in prion-exposed PrP(GPI) *Drosophila* was eIF2 signalling, a pathway that integrates a diverse array of stress-related signals to regulate both global and specific mRNA translation [32]. In addition, prion-exposed PrP(GPI) *Drosophila* were associated with perturbed neuronal signalling pathways including: Dopamine-DARPP32 feedback in cAMP signalling, an important regulator of dopamine/cAMP/PKA signalling [33]; and calcium signalling, a universal second messenger that participates in the transmission of the depolarising signal and contributes to synaptic activity [31]. In prion-exposed PrP(Cyt) *Drosophila* the most statistically relevant canonical pathway was the protein ubiquitination pathway, a principal process responsible for the degradation of intracellular proteins [34]. In addition, these flies were characterised by perturbation of cell cycle control of chromosomal replication and serotonin receptor signalling.

**Table 1:** Top canonical pathways and upstream regulators in prion-exposed PrP transgenic *Drosophila*

(A)			
Fly line	Top canonical pathway	*P*-value	Overlap
PrP(GPI)	EIF2 signalling	2.51 × 10^−10^	14.1% 32/227
	Dopamine-DARPP32 feedback in cAMP signalling	9.73 × 10^−10^	15.9% 26/164
	Calcium signalling	7.54 × 10^−9^	13.6% 28/206
	Mitochondrial dysfunction	4.73 × 10^−8^	14.7% 24/163
	Regulation of eIF4 and p7056K signalling	8.12 × 10^−8^	14.1% 23/163
PrP(Cyt)	Protein ubiquitination pathway	1.61 × 10^−9^	25.6% 62/242
	Cell cycle control of chromosomal replication	4.26 × 10^−9^	44.2% 23/52
	TCA cycle II (eukaryotic)	6.45 × 10^−8^	61.9% 13/21
	EIF2 signalling	1.78 × 10^−7^	24.4% 52/213
	Serotonin receptor signalling	7.22 × 10^−6^	36.6% 15/41
**(B)**			
**Fly line**	**Top upstream regulator**	***P*****-value**	
PrP(GPI)	RICTOR	1.92 × 10^−18^	
	MAPT	2.34 × 10^−17^	
	TP53	8.04 × 10^−12^	
	APP	2.61 × 10^−11^	
	PSEN1	5.02 × 10^−11^	
PrP(Cyt)	E2F1	5.50 × 10^−7^	
	E2F6	4.91 × 10^−6^	
	MAPT	1.07 × 10^−5^	
	ADORA2A	8.98 × 10^−5^	
	PSEN1	1.18 × 10^−3^	

(A) Top canonical pathways; and (B) upstream regulators determined by Ingenuity Pathway Analysis of prion-specific gene transcripts identified in the brains of adult PrP transgenic *Drosophila* following exposure at the larval stage to scrapie-infected sheep brain homogenate. *P*-value probability calculated by the right-tailed Fisher's exact test. The ratio of the number of molecules from the data set that map to the pathway divided by the total number of molecules within the specified canonical pathway is displayed.

Upstream regulator enrichment analysis was used to identify transcriptional regulators responsible, in whole or in part, for the observed prion-induced differential gene expression changes in PrP *Drosophila*, that help characterise the perturbed biological activities occurring in these flies. The data in Table 1B show the top 5 probability-based upstream metabolic pathway regulators perturbed in prion-exposed PrP *Drosophila*. The top-hit upstream regulator in prion-exposed PrP(GPI) *Drosophila* was RICTOR, the rapamycin-insensitive companion of mTOR and defining component of mTORC2 protein complex, an important regulator of synaptic function linked to the control of the actin cytoskeleton [35]. The top-hit upstream regulator in prion-exposed PrP(Cyt) *Drosophila* was the transcription factor E2F1, which can mediate both cell proliferation and TP53/p53-dependent apoptosis [36].

We subsequently inspected the over-represented pathways for genes either up- or down-regulated at different time points in prion-exposed PrP(GPI) *Drosophila* in order to predict when particular cellular functions were enhanced or suppressed following prion infection in the fly. The data in Table 2 show the top 5 probability-ranked functions that were either up- or down-regulated at 5 days or 40 days of age as a consequence of prion-exposure in PrP(GPI) *Drosophila*. At 5 days post hatching, top-ranked up-regulated functions were cell cycle activity and DNA damage regulation, together with functions that control cell cycle progression, namely GADD45 and ataxia telangiectasia mutated (ATM) signalling [37]. The top-ranked down-regulated functions at 5 days of age, were associated with small molecule biochemistry and metabolism of various metabolic cellular components including purine nucleotides. At 40 days of age, the top-ranked up-regulated functions were tyrosine degradation and fatty acid biosynthesis. The top-ranked down-regulated functions at 40 days of age were associated with protein synthesis and mTOR signalling [38].

**Table 2:** Perturbed canonical pathways in prion-exposed PrP(GPI) *Drosophila*

Change	Day 5 Pathways	*P*-value	Day 40 pathways	*P*-value
Up	Cell cycle/DNA damage regulation	5.13 × 10^−6^	Tyrosine degradation I	4.17 × 10^−9^
	GADD45 signalling	1.86 × 10^−5^	Fatty acid activation	1.29 × 10^−3^
	Estrogen-mediated S-phase entry	3.24 × 10^−5^	γ-linolenate biosynthesis II	2.24 × 10^−3^
	Cyclins and cell cycle regulation	5.62 × 10^−5^	Mitochondrial l-carnitine shuttle pathway	2.24 × 10^−3^
	ATM signalling	5.13 × 10^−4^	Glutamate removal from folates	4.17 × 10^−3^
Down	Purine nucleotides	4.07 × 10^−6^	EIF2 signalling	1.00 × 10^−106^
	Tetrahydrobiopterin biosynthesis I	2.19 × 10^−5^	EIF4 regulation & p70S6K signalling	5.01 × 10^−40^
	Tetrahydrobiopterin biosynthesis II	2.19 × 10^−5^	mTOR signalling	2.51 × 10^−34^
	Sorbitol degradation I	2.69 × 10^−3^	Inosine-5′-phosphate biosynthesis II	3.16 × 10^−6^
	Histamine biosynthesis	2.69 × 10^−3^	Purine nucleotides de novo biosynthesis II	1.48 × 10^−5^

Ingenuity Pathway Analysis was used to determine the enriched canonical pathways represented by up- and down-regulated genes in prion-exposed adult PrP(GPI) *Drosophila* following exposure at the larval stage to scrapie-infected sheep brain homogenate. *P*-value probability calculated by the right-tailed Fisher's exact test.

### Identity of cell cycle activity and eIF2 signalling genes perturbed in prion-exposed PrP *Drosophila*

We examined the expression profile of specific genes perturbed in cell cycle activity and protein translation pathways in prion-exposed PrP *Drosophila* compared with control 51D flies. In addition, we used quantitative-PCR (qPCR) in order to validate the expression profile of these pathways identified by RNA-Seq analysis.

**Figure 2.**
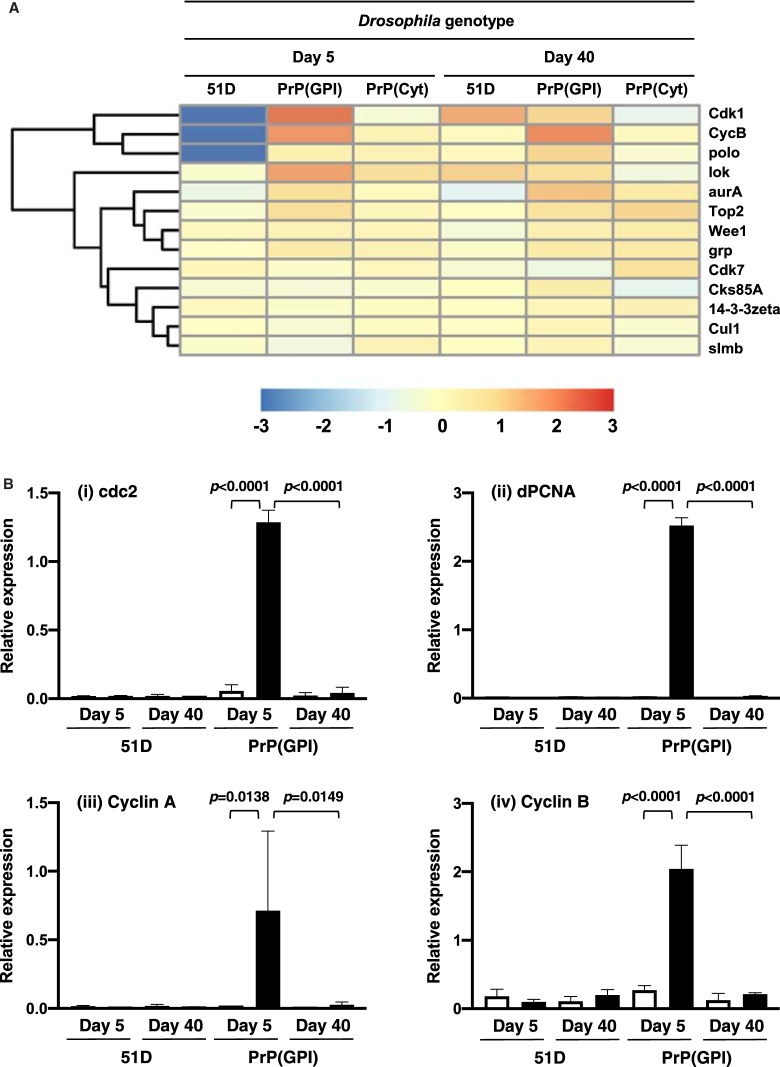
Perturbation of cell cycle:G2/M DNA damage checkpoint regulation pathway genes in prion-exposed *Drosophila*. (**A**) Heatmap showing the effect of prion-infection on cell cycle:G2/M DNA damage checkpoint regulation pathway gene expression in *Drosophila*. Colour indicates the log2-fold difference between prion-exposed and control-treated *Drosophila* gene expression. (**B**) qPCR analysis of cell cycle genes (i) cdc2; (ii) dPCNA; (iii) Cyclin A; and (iv) Cyclin B. Displayed bars represent the relative level of target gene expression ± standard error of the mean, with respect to the reference gene actin. Normal brain homogenate-inoculated (open bars); scrapie-inoculated (filled bars). Statistical analysis between different treatment groups was performed using one-way analysis of variance (ANOVA), together with Tukey honestly significant difference (HSD) for *post hoc* analysis. *P* value probabilities with significance (shown) were identified between relevant PrP(GPI) treatment groups (normal brain homogenate-inoculated and scrapie-inoculated at day 5; or scrapie-inoculated at day 5 and scrapie-inoculated at day 40).

Figure 2 shows the effect of prion infection on cell cycle activity gene expression. Figure 2A shows the heatmap of relative gene expression changes for affected members of cell cycle: G2/M DNA damage checkpoint control pathway in prion-exposed flies. Supplementary Data S3 lists individual affected genes in this pathway. PrP(GPI) *Drosophila* were characterised by up-regulation of genes encoding the Cdk1-cyclin B complex, which serves to trigger mitosis in eukaryotic cells [39] and *Wee1,* a tyrosine kinase Cdk1 regulator [40]. In addition, there was up-regulation of expression of *lok*, a serine/threonine-protein kinase required for checkpoint-mediated cell cycle arrest, activation of DNA repair and apoptosis in response to the presence of DNA double-strand breaks [41]; *Aura*, a mitotic serine/threonine kinase that contributes to the regulation of cell cycle progression [42]; *TOP2*, a topoisomerase II that controls topological states of DNA by transient breakage and subsequent re-joining of DNA strands [43]; *polo*, a Serine/threonine-protein kinase that functions throughout cell cycle M phase [44]; and *grp*, a Serine/threonine-protein kinase required for checkpoint-mediated cell cycle arrest and activation of DNA repair in response to the presence of DNA damage or un-replicated DNA [45]. These changes in cell cycle gene expression were seen in prion-exposed PrP(GPI) *Drosophila* aged 5 days old and 40 days old. In contrast, prion-exposed PrP(Cyt) only showed mild up-regulation of cell cycle activity genes at 40 days of age.

We used quantitative PCR (qPCR) to confirm the early up-regulation of genes associated with cell cycle activity in prion-exposed PrP(GPI) *Drosophila*. The data in Figure 2B show that representative cell cycle genes including cdc2, dPCNA, Cyclin A and Cyclin B were up-regulated in prion-exposed PrP(GPI) *Drosophila* at 5 days of age.

**Figure 3.**
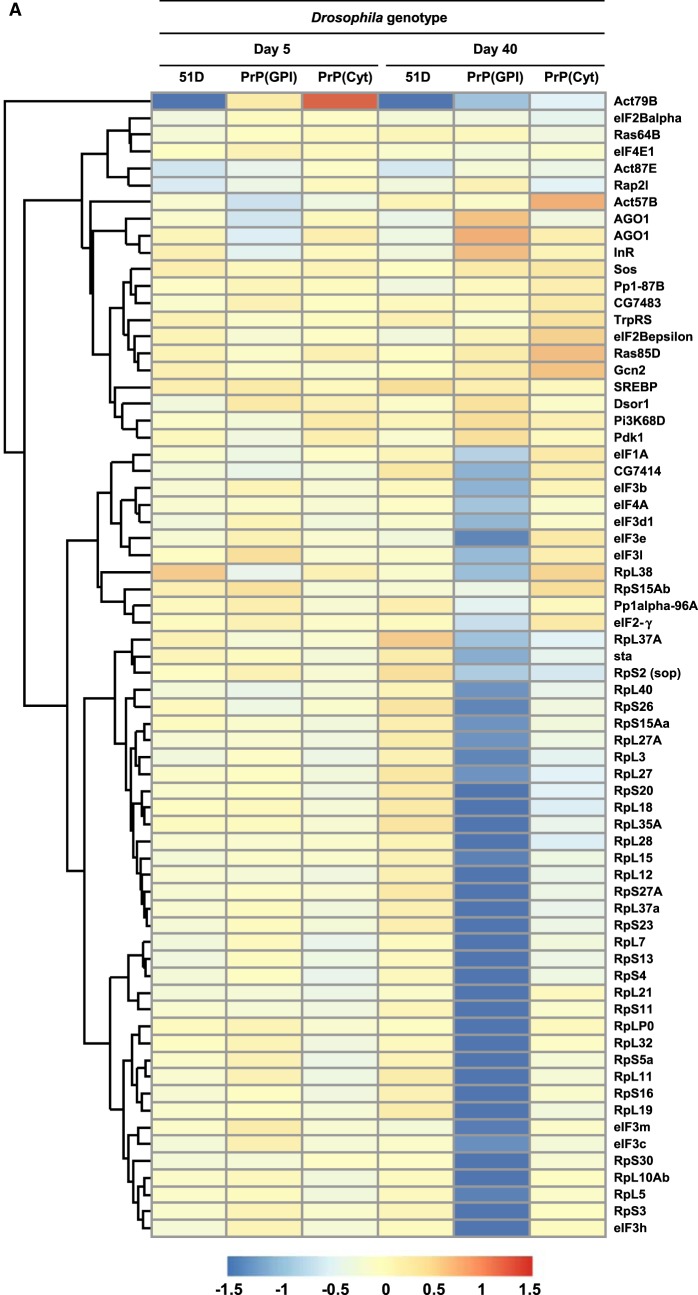

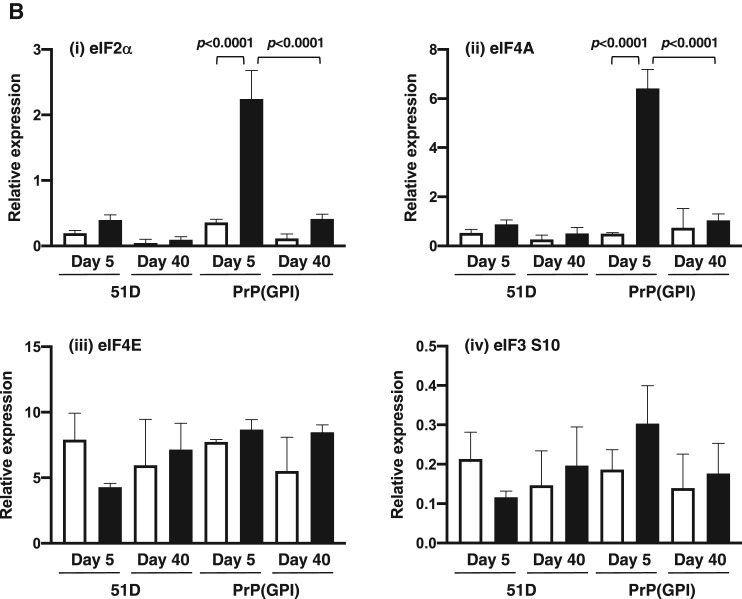
Perturbation of eIF2 signalling cell pathway genes in prion-exposed *Drosophila*. (**A**) Heatmap showing the effect of prion-infection on eIF2 signalling cell pathway gene expression in *Drosophila*. Colour indicates the log2-fold difference between prion-exposed and control-treated *Drosophila* gene expression. (**B**) qPCR analysis of protein translation genes (i) eIF2**α**; (ii) eIF4A; (iii) eIF4E; (iv) and eIF3 S10. Displayed bars represent the relative level of target gene expression ± standard error of the mean, with respect to the reference gene actin. Normal brain homogenate-inoculated (open bars); scrapie-inoculated (filled bars). Statistical analysis between different treatment groups was performed using one-way analysis of variance (ANOVA), together with Tukey honestly significant difference (HSD) for *post hoc* analysis. *P* value probabilities with significance (shown) were identified between relevant PrP(GPI) treatment groups (normal brain homogenate-inoculated and scrapie-inoculated at day 5; or scrapie-inoculated at day 5 and scrapie-inoculated at day 40).

Figure 3 shows the effect of prion infection on eIF2 gene expression in *Drosophila*. Figure 3A shows the heatmap of relative gene expression changes for affected members of the eIF2 signalling pathway [32] of prion-exposed *Drosophila*. Supplementary Data S4 lists the individual affected genes in this pathway. The most significant changes in prion-exposed PrP transgenic *Drosophila* gene expression were seen in flies aged 40 days, with up-, and most prominently, down-regulation of specific transcripts. Prion-exposed PrP(GPI) *Drosophila* at 40 days of age were characterised by up-regulation of various eIF2 signalling genes including: *Gcn2*, an integrated stress response (ISR) protein kinase that phosphorylates eIF2α [46]; *AG01*, an argonaute protein that participates in RNA-mediated gene silencing (RNAi) by the RNA-induced silencing complex (RISC) [47]; *InR*, an insulin-like receptor protein that plays a role in life-span determination [48]; *Sos*, which functions in signalling pathways initiated by the ‘*sevenless*’ and epidermal growth factor receptor tyrosine kinases [49]; *Dsor1*, the mitogen-activated protein kinase kinase 1 (MAP2KK1) [50] that phosphorylates MAP kinase, a member of the extracellular signal-regulated kinases (ERKs) that serve to regulate multiple biochemical signalling pathways; *Pi3K68D*, a phosphoinositide-3-kinase that stimulates the generation of phosphatidylinositol 3,4,5-trisphosphate (PIP3) [51]; and *Pdk1*, a Serine/threonine kinase which acts as a master kinase, phosphorylating and activating a subgroup of protein kinases involved in signal transduction [52]. Prion-exposed PrP(GPI) *Drosophila* at 40 days of age were also characterised by down-regulation of a multitude of genes that encoded either 40S and 60S ribosomal subunit proteins, or genes that encoded components of various translational initiation cofactors, including eIF1, eIF2, eIF3 and eIF4. The data in Figure 3A also show that prion-exposed PrP(Cyt) at 40 days of age were characterised by perturbation of the eIF2 signalling pathway with up-regulation of various genes including: *Gcn2* [46]; eIF2Bε, the guanine nucleotide exchange factor for eIF2 [53]; *Sos*, [49]; and Ras85D, which contributes to the regulation of cell division [54]. Prion-exposed PrP(Cyt) *Drosophila* also showed down-regulation of eIF2 signalling at day 40 although the range and magnitude of the response was less than that seen in similarly treated PrP(GPI) *Drosophila*.

We confirmed down-regulation of protein translation gene expression in prion-exposed PrP(GPI) *Drosophila* by qPCR. The data in Figure 3B show that eIF2α and eIF4A gene expression was down-regulated in a statistically relevant manner in prion-exposed PrP(GPI) *Drosophila* at 40 days of age, whereas eIF4E and eIF3 S10 gene expression showed down-regulation but was not statistically significant.

### Mitochondrial dysfunction is the principal toxicity pathway in prion-exposed PrP *Drosophila*

Pathway analysis was also used to predict potential toxicity pathways responsible for the phenotypic response observed in prion-exposed PrP *Drosophila*. The data in Table 3 show that mitochondrial dysfunction [55], which is characterised by a loss of efficiency in the electron transport chain (ETC) and reductions in the synthesis of adenosine-5′-triphosphate (ATP), was the top toxicity pathway in both PrP(GPI) and PrP(Cyt) prion-exposed *Drosophila* with 20.9% and 13.6% overlap of the differentially expressed genes and pathway genes, respectively.

**Table 3:** Mitochondrial dysfunction identified as top toxicity pathway in prion-exposed *Drosophila*

Fly line	Top toxicity list	*P*-value	Overlap
VRQ(GPI)	Mitochondrial dysfunction	6.89 × 10^−4^	20.9% 33/158
	NRF-mediated oxidative stress response	1.36 × 10^−3^	19.0% 40/210
	PRAR/RXR activation	2.17 × 10^−2^	17.3% 29/168
	CAR/RXR activation	2.37 × 10^−2^	30.0% 6/20
	Cell cycle: G2/M DNA damage checkpoint regulation	3.24 × 10^−2^	21.6% 11/51
VRQ(Cyt)	Mitochondrial dysfunction	8.28 × 10^−8^	13.6% 24/176
	NRF-mediated oxidative stress response	1.67 × 10^−5^	9.9% 25/252
	Cell cycle: G2/M DNA damage checkpoint regulation	1.77 × 10^−4^	17.0% 9/53
	Long term renal injury & anti-oxidative response panel (rat)	4.08 × 10^−4^	27.8% 5/18
	PRAR/RXR activation	1.10 × 10^−3^	9.0% 17/189

Mitochondrial dysfunction was identified by Ingenuity Pathway Analysis as the top toxicity pathway in prion-exposed PrP(GPI) and PrP(Cyt) *Drosophila*. *P*-value probability calculated by the right-tailed Fisher's exact test. The ratio of the number of molecules from the data set that map to the pathway divided by the total number of molecules within the specified canonical pathway is displayed.

**Figure 4.**
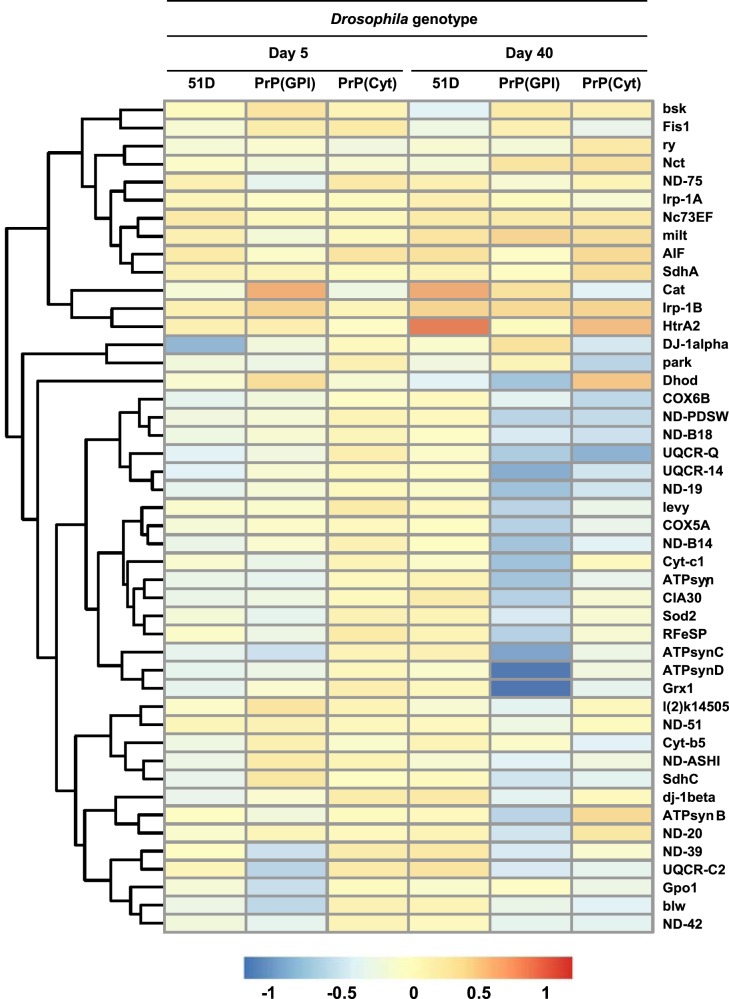
Perturbation of mitochondrial dysfunction pathway genes in prion-exposed *Drosophila*. Heatmap showing the effect of prion-infection on mitochondrial dysfunction pathway gene expression in *Drosophila*. Colour indicates the log2-fold difference between prion-exposed and control-treated *Drosophila* gene expression.

Mitochondrial dysfunction pathway genes differentially expressed in prion-exposed *Drosophila* are shown in the heatmap displayed in Figure 4. Supplementary Data S5 lists individual genes in this pathway. Prion-exposed PrP(GPI) *Drosophila* at 5 days and 40 days of age were characterised by down-regulation of genes involved in the mitochondrial ETC including: *ND*-*20*, -*39*, -*42*, -*51*, *ND-PDSW*, *ND-ASHI* and NADH dehydrogenase, components of mitochondrial respiratory chain complex I; *SdhC*, a component of mitochondrial respiratory chain complex II; Cytochrome b-c1 complex subunit *Rieske*, a component of mitochondrial respiratory chain complex III; *COX5A*, *levy* and *COX6B*, components of cytochrome c oxidase/Complex IV of mitochondrial ETC; *ATPSynB*, *ATPSynC*, *ATPSynD*, and *blw,* components of the inner mitochondrial membrane F1F0 ATP synthase [55]. In addition, prion-exposed PrP(GPI) *Drosophila* were characterised by down-regulation of genes that encode the antioxidants *Sod2* and *Grx1*/Glutaredoxin 1 that participate in redox homeostasis [56]. The data in Figure 4 also show prion-exposed PrP(Cyt) *Drosophila* were characterised by down-regulation of mitochondrial function genes, which was evident in 30 day old flies, albeit to a lesser extent than in PrP(GPI) *Drosophila*. In addition to down-regulation of mitochondrial transport genes in PrP(Cyt) *Drosophila*, *DJ-1α* and *park*, the fly orthologues of the human genes *PARK7* and *PRKN* were down-regulated. *PRKN* encodes a ubiquitin ligase protein that is implicated in the human protein misfolding neurodegenerative condition Parkinson's disease [57].

**Figure 5.**
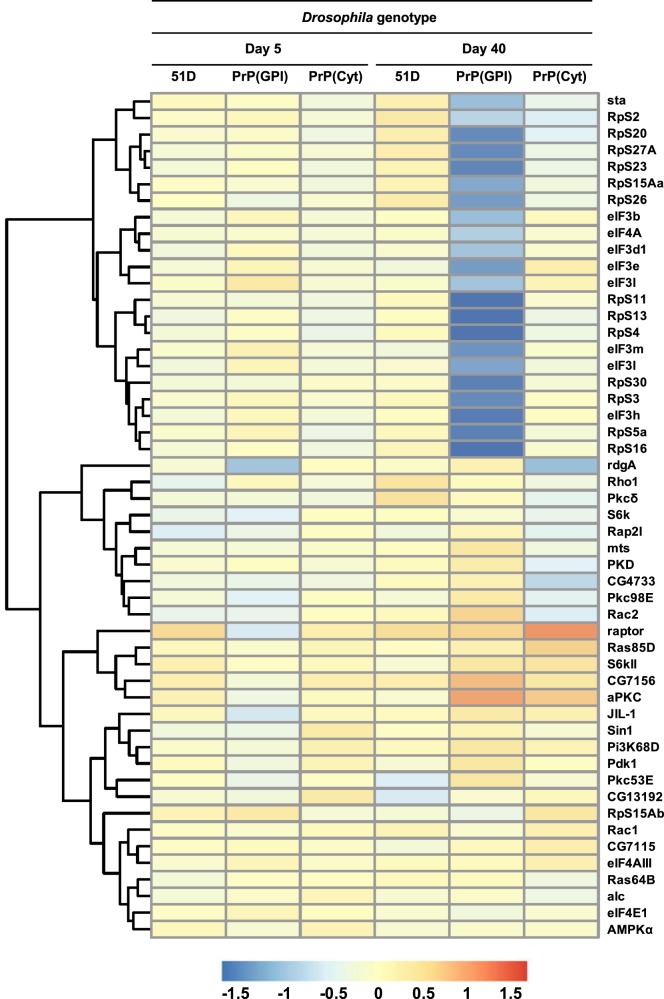
Perturbation of mTOR signalling pathway genes in prion-exposed *Drosophila*. Heatmap showing the effect of prion-infection on mTOR signalling cell pathway gene expression in *Drosophila*. Colour indicates the log2-fold difference between prion-exposed and control-treated *Drosophila* gene expression.

A key regulator of cellular processes and pathways is the mTOR signalling pathway [58]. Disturbances in mTOR signalling in the brain affects multiple pathways including cellular metabolism, mitochondrial function and autophagy. The heatmap data in Figure 5 show that prion-exposed PrP(GPI) *Drosophila* at 5 days of age were characterised by mild changes in expression for some genes of the mTOR pathway, listed in Supplementary Data S6, but at 40 days of age there were marked changes in gene expression. Prion-exposed PrP(GPI) *Drosophila* at 40 days of age were characterised by up-regulation of *Dsor1* [50]; *Lk6*, *a* serine/threonine-protein kinase that interacts with mitogen-activated protein kinase 1 MAPk1 / ERK2 [59]; Pkc98E, a regulatory protein kinase activated by diacylglycerol or Ca^2+^ [60]. In addition, at 40 days of age prion-exposed PrP(GPI) *Drosophila* were characterised by down-regulation of a number of mTOR pathway genes that encoded translational initiation cofactors including eIF3, or either 40S and 60S ribosomal subunit proteins.

## Discussion

Here we have performed RNA-Seq-based transcriptome analysis of prion-exposed PrP transgenic *Drosophila* in order to probe the identity of cellular and molecular pathways associated with prion-induced neurotoxicity. Adult *Drosophila* transgenic for pan neuronal expression of PrP(GPI), previously exposed to infectious prions at the larval stage, first displayed up-regulation of genes associated with cell cycle control and the DNA damage response (DDR), and subsequently down-regulation of genes associated with initiation of protein synthesis, namely eIF2 signalling and its regulatory pathway mTOR (mechanistic target of rapamycin). Furthermore, we identified mitochondrial dysfunction as the major toxicity pathway in prion-exposed PrP transgenic *Drosophila*. Our analysis, in a unique invertebrate model of transmissible mammalian prion disease, indicates that aberrant cell cycle activity, perturbation of protein synthesis and mitochondrial dysfunction are principal dysregulated cellular systems involved in prion-induced neurotoxicity.

We identified up-regulation of genes involved with cell cycle activity and DDR in 5 day old prion-exposed PrP(GPI) *Drosophila* after their exposure to scrapie material at the larval stage. At this time these prion-exposed flies do not show any significant locomotor defect [24]. The DDR functions in surveillance and repair of DNA lesions during cell cycle progression in order to maintain integrity of the cellular genome between successive generations. The G2/M interface DDR checkpoint control allows for DNA damage during replication to be repaired prior to mitosis. During DDR, chromatin undergoes transient disaggregation at the sites of DNA lesion to facilitate access of repair and cell cycle checkpoint proteins [61–63]. Since open chromatin is evident in regions of actively transcribed DNA, heterochromatin relaxation in response to DDR can trigger aberrant gene expression of normally silenced regions of the genome. In this context, it has been shown that wide-spread loss of heterochromatin occurs in *Drosophila* and mouse tauopathy models, and human Alzheimer's disease, and that this is associated with aberrant gene expression in CNS neurons [64]. Usually, post mitotic neurons do not participate in cell cycle activity and to do so is considered to be detrimental to these cells [65]. In this context, post mitotic neurons may have the potential to revert to a de-differentiated state, which might be linked to activation of apoptotic pathways and concomitant neurodegeneration [66,67].

Our finding of cell cycle activity and DDR involvement in prion-mediated neurotoxicity is supported by observations from other studies. For example, nuclear accumulation of proliferating cell nuclear antigen (PCNA) and phosphorylated histone H2A.X proteins, which are indicative of DNA replication and/or repair in other cell types, have been detected in CNS neurons of mice used to model familial CJD and FFI prion diseases [68]. In addition, the brains of scrapie-affected hamsters show evidence of cell cycle activity with an increase in the proteins polo-like kinase (PLK) 1 and cyclin B1, and a decrease in PLK3 and Cdc25C [69]. In addition, prion infectivity experiments *in vivo* have shown that mice deficient in base-excision repair activity displayed an accelerated clinical course of prion disease compared with wild type animals [70]. Cell cycle activity and DDR may be fundamental to protein misfolding neurodegenerative diseases in general since these cellular activities have been reported in other such conditions [71].

We identified a down-regulation of eIF2 signalling gene expression in 40 day old prion-exposed PrP(GPI) *Drosophila*, previously exposed to scrapie material at the larval stage. Neurons are highly dependent upon sustained and efficient mRNA translation in order to undergo neurotransmitter release and exhibit synaptic plasticity. Prion-exposed PrP(GPI) *Drosophila* showed a dramatic down-regulation of multiple genes of the eIF2 signalling pathway, including those that encoded translational initiation factor proteins, and 40S and 60S ribosomal proteins. Since eIF2 signalling is a major regulator of initiation of mRNA translation [32], down-regulation of this pathway was indicative of suppression of protein synthesis in prion-exposed *Drosophila*. The down-regulation of eIF2 signalling in prion-exposed PrP(GPI) *Drosophila* correlated with the accelerated decline in locomotor activity seen in these flies at this particular time point.

Important regulators of eIF2 signalling are the ISR [72] or the unfolded stress response [73], which inhibit general protein synthesis through eIF2α phosphorylation. EIF2α phosphorylation leads to sequestration of the eIF2α guanine nucleotide exchange factor eIF2B, which in turn prevents exchange of GTP for GDP thereby reducing availability of the initiation ternary complex eIF2-GTP-Met-tRNAi. De-phosphorylation of eIF2α occurs via the phosphatase complexes GADD34 or CREP. Our observation that the fly gene *Gcn2*, which encodes a kinase that phosphorylates eIF2α, was up-regulated in prion-exposed PrP(GPI) *Drosophila* is indicative of activation of the ISR in these flies. This observation correlates with studies in cells [21] and mice [22] that show an ongoing prion infection triggers activation of the PERK/eIF2α branch of the unfolded protein response that in turn leads to a block of protein translation. Increased levels of phosphorylated eIF2α are evident in other protein misfolding neurodegenerative disorders [74], which suggests that reduced rates of translation initiation may contribute to the pathology seen in these conditions.

A second major regulator of the eIF2 signalling pathway is mTOR which mediates phosphorylation of the initiation factors eIF4G, eIF4B and the initiation factor inhibitor eIF4E-binding protein (4E-BP) [75]. 4E-BP competes with eIF4G for interaction with eIF4E and mTOR-mediated de-phosphorylation of 4E-BP causes the protein to detach from eIF4E, relieving inhibition of eIF2-associated guanidine exchange [76]. In addition, mTOR is also critically involved in the regulation of autophagy, which functions in order to avoid the accumulation of aberrantly folded toxic protein aggregates, or organelles, that may contribute to protein misfolding conditions, including prion diseases [77]. We showed here that 40 day old prion-exposed PrP(GPI) *Drosophila* were characterised by down-regulation in mTOR gene expression. Down-regulation of mTOR signalling in prion-exposed neurons may therefore not only affect protein synthesis and autophagy, but also mitochondrial function [78]. Disturbances in autophagy are reported in various human pathologies including neurodegenerative disorders [79].

We identified mitochondrial dysfunction as the principal toxicity pathway in 40 day old prion-exposed PrP(GPI) *Drosophila.* Impaired mitochondrial function can occur through changes in mitochondrial dynamics via fission and fusion, alteration in the concentration and activity of ETC components, or oxidative stress [80]. We showed that prion-exposed PrP(GPI) *Drosophila* were characterised by down-regulation of genes encoding principal components of the ETC including NADH dehydrogenase, ATP synthase, and various antioxidants including superoxidase dismutase-2 and Glutaredoxin 1. Neurons, in particular their synaptic regions, are vulnerable to mitochondrial dysfunction because of the need to satisfy the large energy demands of synaptic development, transmission, and plasticity. In addition, mitochondria are involved in the buffering of intracellular calcium and the storage of pro-apoptotic mediators. Loss of mitochondrial membrane integrity leads to leakage of these mediators and cell death via necrosis or apoptosis. Mitochondrial dysfunction has been shown in hamster [81] and mouse [82,83] models of prion disease. In addition, down-regulation of mRNA and protein levels of mitochondrial proteins have been reported in post-mortem tissues of CJD patients [84]. Mitochondrial dysfunction has been observed in other protein misfolding neurodegenerative diseases, including Alzheimer's disease, Parkinson's disease, Huntington's disease, and amyotrophic lateral sclerosis [55].

The cellular functions and biochemical processes that we have identified as perturbed in prion-exposed PrP(GPI) *Drosophila* would seem to be directly relevant to prion disease for the following reasons. Firstly, the pathways identified occur in a fly model of transmissible mammalian prion disease that is characterised by authentic prion replication [24]. Secondly, our studies here have used *Drosophila* transgenic for pan neuronal expression of PrP. Since prion-induced toxicity only occurs in cells that express PrP, the changes in gene expression profile we have observed reflect neurotoxicity. Third, the gene expression profile of prion-exposed PrP(GPI) *Drosophila* identified here was specific to this fly line and was not seen in similarly treated PrP(Cyt) *Drosophila*, which indicates the observed phenotype was not due to non-specific PrP-mediated toxicity. Collectively, our studies are consistent with the disturbance of multiple cellular and biochemical network systems, namely aberrant cell cycle activity and DDR, repression of protein synthesis and mitochondrial dysfunction, during prion-induced neurotoxicity.

**Figure 6.**
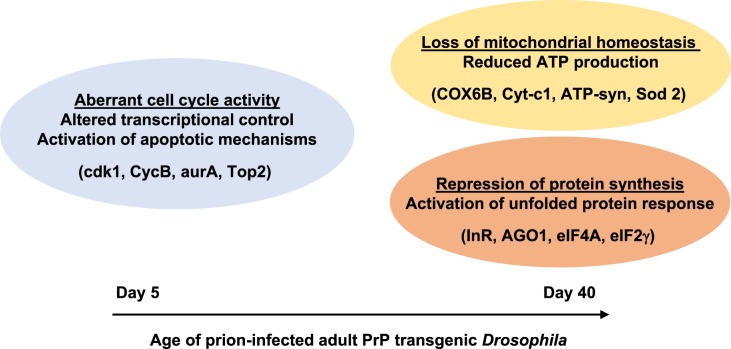
Model for prion-induced neurotoxicity in PrP transgenic *Drosophila*. Proposed major cellular events, together with representative participating genes (shown in brackets), associated with prion-induced neurotoxicity in PrP transgenic *Drosophila*.

Our observations, which are supported by transcriptomic studies in prion-infected mammalian hosts [85–87] are consistent with perturbation of multiple cellular and biochemical network systems during prion-induced neurotoxicity. Each of these prion-perturbed systems displays its own unique dynamic change in constituent gene expression level and does so in a temporal manner. This suggests that dynamic perturbations of gene expression in systems and processes associated with prion-induced neurotoxicity occurs in a specific order. Accordingly, we propose that following initial prion infection at the larval stage, that subsequent PrP^Sc^ accumulation within neurons has an adverse effect upon critical cellular processes, that in turn leads to a genotoxic effect with resultant dysregulated gene expression and aberrant cell cycle activity coupled with the activation of apoptotic mechanisms. We further propose that these early prion-induced events in neurons drive a loss of mitochondrial homeostasis and a repression of protein synthesis as neurons attempt to accommodate the cellular stress associated with PrP misfolding. This hypothesis is summarised in Figure 6. We cannot yet differentiate whether the proposed loss of mitochondrial homeostasis, which will invariably be accompanied by a reduction in ATP production, is the stimulus for repression of protein synthesis, or whether loss of protein synthesis drives loss of normal mitochondrial status. We are now in a position to test these possibilities through our use of *Drosophila*, a genetically well defined tractable experimental host amenable to silencing and overexpression of specific genes, to probe the role of mitochondrial dysfunction in prion-induced neurotoxicity, a cellular function increasingly implicated in protein misfolding-induced neurodegeneration [55,88].

## Open Access

Open access for this article was enabled by the participation of University of Cambridge in an all-inclusive *Read & Publish* pilot with Portland Press and the Biochemical Society under a transformative agreement with JISC.

## Abbreviations

ANOVA: analysis of variance
ATM: ataxia telangiectasia mutated
ATP: adenosine-5′-triphosphate
BSE: bovine spongiform encephalopathy
CJD: Creutzfeldt–Jakob disease
DDR: DNA damage response
ERKs: extracellular signal-regulated kinases
ETC: electron transport chain
HSD: honestly significant difference
IPA: Ingenuity Pathway Analysis
ISR: integrated stress response
MAP2KK1: mitogen-activated protein kinase kinase 1
MGA: Multiple Genome alignment
PCNA: proliferating cell nuclear antigen
PI: performance index
PIP3: phosphatidylinositol 3,4,5-trisphosphate
PK: Proteinase K
PLK: polo-like kinase
RISC: RNA-induced silencing complex
RT: reverse transcription

## Supplementary Material

Supplementary Datas S1-S6
