# Supplementary material for: Transcriptional signature of prion-induced neurotoxicity in a Drosophila model of transmissible mammalian prion disease
Source: Biochem J. 2020 Feb 28;477(4):833–52. doi: 10.1042/BCJ20190872 (PMC7054746; doi:10.1042/BCJ20190872)
Supplement: Supplementary Datas S1-S6 [file BCJ-477-833-s1.pdf]

Supplementary data legends

Supplementary data 1. Sequences of qPCR primers
Sequences of primers used in qPCR to detect cell cycle and protein translation gene expression level.

Supplementary data 2. Prion-exposed PrP transgenic *Drosophila* show accelerated decline in locomotor activity
(A) PrP(GPI); (B) PrP(Cyt); or (C) control 51D *Drosophila* crossed with the *Elav-GAL4* driver line were assessed for locomotor activity at various time points after hatching by a negative geotaxis climbing assay following exposure at the larval stage to scrapie-infected sheep (red symbols and red line) or scrapie-free (blue symbols and blue line) sheep brain homogenate. The mean performance index is shown for three groups of n=15 flies of each genotype per time point (45 flies in total for each genotype at the start of the experiment). Statistical analysis of the scrapie-infected and scrapie-free linear regression plots for each fly line was performed by the unpaired samples *t* test. (Data modified from original figure in Thackray et al. 2014, reference 26, doi: https://doi.org/10.1042/BJ20140129).

Supplementary data 3. Cell cycle:G2/M DNA damage checkpoint regulation pathway genes
List of *Drosophila* and orthologous human genes, together with their function, involved in the cell cycle:G2/M DNA damage checkpoint regulation pathway.

Supplementary data 4. eIF2 signalling pathway genes
List of *Drosophila* and orthologous human genes, together with their function, involved in the eIF2 signalling pathway.

Supplementary data 5. Mitochondrial dysfunction pathway genes
List of *Drosophila* and orthologous human genes, together with their function, involved in the mitochondrial dysfunction pathway.

Supplementary data 6. mTOR signalling pathway genes
List of *Drosophila* and orthologous human genes, together with their function, involved in the mTOR signalling pathway.

Supplementary data 1. Sequences of qPCR primers

Gene	Primer direction	Primer sequence
cdc2	Forward	CGTGGTGTATAAGGGTCGCA
cdc2	Reverse	ACGAAATTTCTCTGATCGCGG
dPCNA	Forward	GTCAAGCCACCATCCTGAAGA
dPCNA	Reverse	CCTGTAGCTGAATGCCGGAG
Cyclin A	Forward	AAGAGTCAAGGAGCTTCCGC
Cyclin A	Reverse	TGTTTCTTCTCGCTCTCCCG
Cyclin B	Forward	CACTGTAGAACCCACTAAAGTTACA
Cyclin B	Reverse	GGTCAGCGACTTCTTCGACA
eIF2α	Forward	GCACCGCCACTCTATGTAATG
eIF2α	Reverse	TCCATCGTACTCGCTGGTCT
eIF4A	Forward	ATCATTCCCTGTGTGAGGGG
eIF4A	Reverse	ATGCTCGTGTCGATTTGCTG
eIF4E	Forward	CATGTTCAAAACCAGCGCCC
eIF4E	Reverse	GTTCACCAGTCTCCTGTGGG
eIF3-S10	Forward	TATTGAGGCGGACAAGAGCC
eIF3-S10	Reverse	ATTCAAGCGCACCACTTCAC
Actin	Forward	TTGGATTTACACGTCTGCCGC
Actin	Reverse	TTGTCTACGACCAGGGCTGA

Supplementary data 2. Prion-exposed PrP transgenic *Drosophila show* accelerated decline in locomotor activity

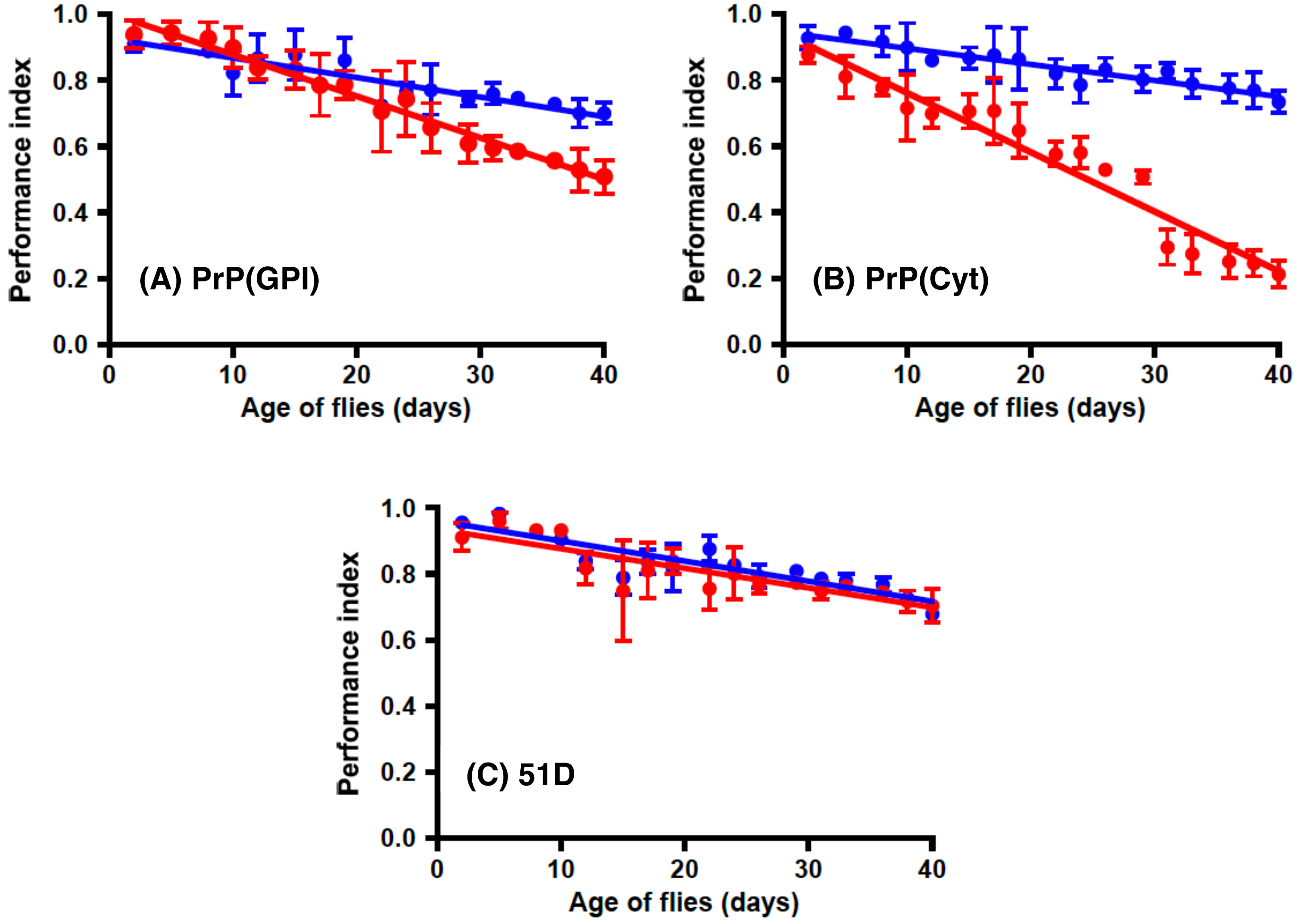

Supplementary data 3. Cell cycle - G2/M DNA damage checkpoint regulation pathway genes

Fly gene number	Fly gene name	Human gene name	Gene product
FBgn0004106	Cdk1	CDK1	Cyclin-dependent kinase 1, activated by cyclins and controls cell cycleprogression
FBgn0000405	CycB	CCNB!	Cyclin B, binds to CDK1 and the resultant complex induces the start of mitosis
FBgn0003124	polo	PLK1	Serine/threonine-protein kinase that functions throughout cell cycle M phase
FBgn0019686	lok	CHEK2	Serine/threonine-protein kinase, mediates DNA double-strand break cell cycle arrest, DNA repair and apoptosis
FBgn0000147	aurA	AURKA	Mitotic serine/threonine kinase that contributes to the regulation of cell cycle progression
FBgn0284220	Top2	TOP2A	Topoisomerase 2, alters the topology of DNA by creating a transient DNA double strand breakage
FBgn0011737	Wee1	WEE1	Non-specific protein-tyrosine kinase required for triggering entry into mitosis
FBgn0261278	grp	CHEK1	Grapes, protein kinase that functions in a cell cycle checkpoint pathway that responds to DNA damage
FBgn0263237	Cdk7	CDK7	Cyclin-dependent kinase 7, activated by the binding to a cyclin and mediates progression through cell cycle.
FBgn0037613	Cks85A	CKS2	Cyclin-dependent kinase subunit 85A, binds cyclin dependent kinases and is essential for their function
FBgn0004907	14-3-3zeta	YWHAZ	Acidic protein isoforms that function in signaling pathways, most prominently Ras/MAPK cascade.
FBgn0015509	Cul1	CUL1	Cullin 1, key assembly factor of SCF (SKP1-CUL1-F-box protein) E3 ubiquitin ligase complexes
FBgn0283468	slmb	BTRC	Supernumerary limb, F-box component of Skp/Cullin/F-box E3 ubiquitin-ligase, promotes proteasome activity

Supplementary data 4. eIF2 signalling pathway genes

Fly gene number	Fly gene name	Human gene name	Gene product
FBgn0000045	Act79B	ACTL7A	Actin protein isoform, structural constituent of cytoskeleton
FBgn0039726	eIF2Balpha	EIF2B1	Eukaryotic translation initiation factor 2B subunit alpha
FBgn0003206	Ras64B	RRAS2	Ras oncogene at 64B
FBgn0015218	eIF4E1	EIF4E	Eukaryotic translation initiation factor 4E1, binds the 7-methyl-guanosine cap structure of mRNA
FBgn0000046	Act87E	ACTRT2	Actin protein isoform, structural constituent of cytoskeleton
FBgn0283666	Rap2l	RAP2C	Ras-associated protein 2-like involved in regulation of proliferation, differentiation, and apoptosis
FBgn0000044	Act57B	ACTRT2	Actin protein isoform, structural constituent of cytoskeleton
FBgn0262739	AGO1	AGO2	Argonaute-1 of miRNA-induced silencing complexes (miRISCs).
FBgn0000711	flw	PPP1CB	Flapwing, serine/threonine protein phosphatase type 1β
FBgn0283499	InR	IGF1R	Insulin-like receptor with tyrosine-protein kinase activity, required for cell survival
FBgn0001965	Sos	SOS1	Ras-bound GDP/ GTP exchange factor involved in tyrosine kinases receptor signalling pathways
FBgn0004103	Pp1-87B	PPP1CA	Serine/threonine-protein phosphatase alpha-2 isoform
FBgn0037573	CG7483	EIF4A3	Eukaryotic translation initiation factor 4A3
FBgn0010803	TrpRS	WARS	Tryptophanyl-tRNA synthetase
FBgn0023512	eIF2Bepsilon	EIF2B5	Eukaryotic translation initiation factor 2B subunit epsilon
FBgn0003205	Ras85D	HRAS	Ras oncogene 85D protein, acts via receptor tyrosine kinases, involved in cell growth and survival
FBgn0019990	Gcn2	EIF2AK4	eIF-2a kinase, activator of integrated stress response, phosphorylates eIF2a to halt protein translation
FBgn0261283	SREBP	SREBF1	Sterol regulatory element binding protein, membrane protein and master-regulator of lipogenesis
FBgn0010269	Dsor1	MAP2K1	Downstream of raf1, a serine/threonine kinase that phosphorylates MAP kinase
FBgn0015278	Pi3K68D	PIK3C2A	Phosphotidylinositol 3 kinase 68D
FBgn0020386	Pdk1	PDPK1	Phosphoinositide-dependent kinase 1
FBgn0026250	eIF1A	EIF1AX	Eukaryotic translation initiation factor 1A
FBgn0037135	CG7414	EIF2A	Eukaryotic translation initiation factor 2A
FBgn0034237	eIF3b	EIF3B	Eukaryotic translation initiation factor 3 subunit b
FBgn0001942	eIF4A	EIF4A1	Eukaryotic translation initiation factor 4A
FBgn0040227	eIF3d1	EIF3D	Eukaryotic translation initiation factor 3 subunit d1
FBgn0025582	eIF3e	EIF3E	Eukaryotic translation initiation factor 3 subunit e
FBgn0036258	eIF3l	EIF3L	Eukaryotic translation initiation factor 3 subunit l, facilitates the 43S pre-initiation complex (43S PIC)
FBgn0040007	RpL38	RPL38	Ribosomal protein L38, 60S component
FBgn0033555	RpS15Ab	RPS15A	Ribosomal protein S15A, 40S component
FBgn0003134	Pp1alpha-96A	PPP1CC	Serine/threonine-protein phosphatase alpha-1 isoform
FBgn0263740	eIF2-γ	EIF2S3	Eukaryotic translation initiation factor 2 subunit- γ
FBgn0261608	RpL37A	RPL37A	Ribosomal protein L37a, 60S component
FBgn0003517	sta	RPSA	Ribosomal protein of ≈30Kda, 40S component
FBgn0004867	RpS2 (sop)	RPS2	Ribosomal protein S2, 40S component
FBgn0003941	RpL40	UBA52	Ribosomal protein L40, 60S component
FBgn0261597	RpS26	RPS26	Ribosomal protein S26, 40S component
FBgn0010198	RpS15Aa	RPS15A	Ribosomal protein S15Aa, 40S component
FBgn0285948	RpL27A	RPL27A	Ribosomal protein L27A, 60S component
FBgn0020910	RpL3	RPL3L	Ribosomal protein L3, 60S component
FBgn0039359	RpL27	RPL27	Ribosomal protein L27A, 60S component
FBgn0019936	RpS20	RPS20	Ribosomal protein S20, 40S component
FBgn0035753	RpL18	RPL18	Ribosomal protein L18, 60S component
FBgn0037328	RpL35A	RPL35A	Ribosomal protein L35A, 60S component
FBgn0035422	RpL28	RPL28	Ribosomal protein L28, 60S component
FBgn0028697	RpL15	RPL15	Ribosomal protein L15, 60S component
FBgn0034968	RpL12	RPL12	Ribosomal protein L12, 60S component
FBgn0003942	RpS27A	RPS27A	Ribosomal protein S27A, 40S component
FBgn0030616	RpL37a	RPL37	Ribosomal protein L37a, 60S component
FBgn0033912	RpS23	RPS23	Ribosomal protein S23, 40S component
FBgn0005593	RpL7	RPL7L1	Ribosomal protein L7, 60S component
FBgn0010265	RpS13	RPS13	Ribosomal protein S13, 40S component
FBgn0011284	RpS4	RPS4Y1	Ribosomal protein S4, 40S component
FBgn0032987	RpL21	RPL21	Ribosomal protein L21, 60S component
FBgn0033699	RpS11	RPS11	Ribosomal protein S11, 40S component
FBgn0000100	RpLP0	RPLP0	Ribosomal protein LP0, 60S component
FBgn0002626	RpL32	RPL32	Ribosomal protein L32, 60S component
FBgn0002590	RpS5a	RPS5	Ribosomal protein S5a, 40S component
FBgn0013325	RpL11	RPL11	Ribosomal protein L11, 60S component
FBgn0034743	RpS16	RPS16	Ribosomal protein S16, 40S component
FBgn0285950	RpL19	RPL19	Ribosomal protein L19, 60S component
FBgn0033902	eIF3m	EIF3M	Eukaryotic translation initiation factor 3 subunit m
FBgn0034258	eIF3c	EIF3CL	Eukaryotic translation initiation factor 3 subunit c
FBgn0038834	RpS30	FAU	Ribosomal protein S30, 40S component
FBgn0036213	RpL10Ab	RPL10A	Ribosomal protein L10Ab, 60S component
FBgn0064225	RpL5	RPL5	Ribosomal protein L5, 60S component
FBgn0002622	RpS3	RPS3	Ribosomal protein S3, 40S component
FBgn0022023	eIF3h	EIF3H	Eukaryotic translation initiation factor 3 subunit h

Supplementary data 5. Mitochondrial dysfunction pathway genes

Fly gene number	Fly gene name	Human gene name	Gene product
FBgn0000229	bsk	MAPK	Mitogen-activated protein kinase
FBgn0039969	Fis1	FIS1	Functions downstream of Pink1 and upstream of Drp1 to regulate mitochondrial fission
FBgn0003308	ry	XDH	Xanthine dehydrogenase, involved in the oxidative metabolim of purines
FBgn0039234	Nct	NCSTN	Subunit of g-secretase complex that catalyzes intramembrane cleavage of integral membrane proteins such as Notch
FBgn0017566	ND-75	NDUFS1	NADH dehydrogenase (ubiquinone) 75 kDa subunit
FBgn0024958	Irp-1A	ACO1	Iron regulatory protein 1A, a cytosolic aconitase involved in iron sensing
FBgn0010352	Nc73EF	OGDHL	Neural conserved at 73EF, isoform I, an oxoglutarate dehydrogenase (succinyl-transferring).
FBgn0262872	milt	HAP1	Trafficking kinesin-binding protein, required for kinesin-mediated axonal transport of mitochondria to nerve terminals
FBgn0031392	AIF	AIFM1	Apoptosis inducing factor that participates in cell death and oxidative phosphorylation in mitochondria
FBgn0261439	SdhA	SDHA	Succinate dehydrogenase subunit A, encodes an FAD-containing subunit of the succinate dehydrogenase complex.
FBgn0000261	Cat	CAT	Catalase, catalyzes the decomposition of hydrogen peroxide
FBgn0024957	Irp-1B	ACO1	Iron regulatory protein 1B, cytosolic aconitase involved in iron ion homeostasis
FBgn0038233	HtrA2	HTRA2	Mitochondrial serine protease that induces cell death by inhibition of anti-apoptotic proteins
FBgn0033885	DJ-1alpha	PARK7	Mitogen-activated protein kinase, acts as an oxidative stress sensor to protect against oxidative stress
FBgn0041100	park	PRKN	RBR-type E3 ubiquitin transferase, functions in the regulation of mitochondrial fission
FBgn0000447	Dhod	DHODH	Dihydroorotate dehydrogenase, an inner mitochondrial membrane protein involved in de novo pyrimidine biosynthesis
FBgn0031066	COX6B	COX6B2	Cytochrome c oxidase subunit 6B
FBgn0021967	ND-PDSW	NDUFB10	NADH dehydrogenase (ubiquinone) PDSW subunit
FBgn0030605	ND-B18	NDUFB7	NADH dehydrogenase (ubiquinone) B18 subunit
FBgn0036728	UQCR-Q	UQCRQ	Ubiquinol-cytochrome c reductase ubiquinone-binding protein
FBgn0030733	UQCR-14	UQCRB	Ubiquinol-cytochrome c reductase 14 kDa subunit
FBgn0035046	ND-19	NDUFA8	NADH dehydrogenase (ubiquinone) 19 kDa subunit
FBgn0034877	levy	COX6A2	Exhibits enzyme regulator activity, contributes to cytochrome-c oxidase activity
FBgn0019624	COX5A	COX5A	Cytochrome c oxidase subunit 5A
FBgn0033570	ND-B14	NDUFA6	NADH dehydrogenase (ubiquinone) B14 subunit
FBgn0035600	Cyt-c1	CYC1	Cytochrome c1
FBgn0020235	ATPsyn γ	ATP5F1C	ATP synthase γ subunit
FBgn0039689	CIA30	NDUFAF1	Mitochondrial complex I intermediate-associated protein 30, a chaperone required for assembly of complex I
FBgn0010213	Sod2	SOD2	Superoxide dismutase 2 (Mn) (Sod2), an enzyme that detoxifies superoxide radicals in mitochondria
FBgn0021906	RFeSP	UQCRFS1	Rieske iron-sulfur protein, predicted to contribute to ubiquinol-cytochrome-c reductase activity
FBgn0039830	ATPsynC	ATP5MC2	ATP synthase, subunit C
FBgn0016120	ATPsynD	ATP5PD	ATP synthase, subunit D
FBgn0036820	Grx1	GLRX2	Glutaredoxin 1, facilitates mitochondrial redox homeostasis during oxidative stress-induced apoptosis
FBgn0021856	l(2)k14505	ATPAF2	ATP synthase mitochondrial F1 complex assembly factor 2
FBgn0031771	ND-51	NDUFV1	NADH dehydrogenase (ubiquinone) 51 kDa subunit, component of mitochondrial respiratory chain Complex I
FBgn0264294	Cyt-b5	CYB5B	Cytochrome b5, a membrane-bound hemoprotein that functions in redox reactions
FBgn0029888	ND-ASHI	NDUFB8	NADH dehydrogenase (ubiquinone) ASHI subunit of the mitochondrial membrane respiratory chain Complex I
FBgn0037873	SdhC	SDHC	Subunit of succinate dehydrogenase located in mitochondrial electron transport chain complex II
FBgn0039802	dj-1beta	PARK7	Protein Dj-1 β, involved in protection against reactive oxygen species
FBgn0010217	ATPsyn β	ATP5F1B	β-subunit of the inner mitochondrial membrane F1F0 ATP synthase
FBgn0030718	ND-20	NDUFS7	NADH dehydrogenase (ubiquinone) 20 kDa subunit, a subunit of mitochondrial respiratory complex I
FBgn0037001	ND-39	NDUFA9	NADH dehydrogenase (ubiquinone) 39 kDa subunit, a subunit of mitochondrial respiratory complex I
FBgn0250814	UQCR-C2	UQCRC2	Ubiquinol-cytochrome c reductase core protein 2
FBgn0022160	Gpo1	GPD2	Glycerophosphate oxidase 1, mitochondrial inner membrane protein with glycerol-3 phosphate dehydrogenase activity
FBgn0011211	blw	ATP5F1A	α-subunit of the inner mitochondrial membrane F1F0 ATP synthase
FBgn0019957	ND-42	NDUFA10	NADH dehydrogenase (ubiquinone) 42 kDa subunit, component of mitochondrial respiratory chain Complex I

Supplementary data 6. mTOR signalling pathway genes

Fly gene number	Fly gene name	Human gene name	Gene product
FBgn0003517	sta	RPSA	40S ribosomal protein SA, required for the assembly and/or stability of the 40S ribosomal subunit
FBgn0004867	RpS2	RPS2	Ribosomal protein S2, 40S component
FBgn0019936	RpS20	RPS20	Ribosomal protein S20, 40S component
FBgn0003942	RpS27A	RPS27A	Ribosomal protein S27A, 40S component
FBgn0033912	RpS23	RPS23	Ribosomal protein S23, 40S component
FBgn0010198	RpS15Aa	RPS15A	Ribosomal protein S15Aa, 40S component
FBgn0261597	RpS26	RPS26	Ribosomal protein S26, 40S component
FBgn0034237	eIF3b	EIF3B	Eukaryotic translation initiation factor 3B
FBgn0001942	eIF4A	EIF4A2	Eukaryotic translation initiation factor 4A, RNA helicase involved in mRNA cap recognition
FBgn0040227	eIF3d1	EIF3D	Eukaryotic translation initiation factor 3 subunit d1, mRNA cap- binding protein
FBgn0025582	eIF3e	EIF3E	Eukaryotic translation initiation factor 3 subunit e
FBgn0036258	eIF3l	EIF3L	Eukaryotic translation initiation factor 3 subunit l, facilitates the 43S pre-initiation complex
FBgn0033699	RpS11	RPS11	Ribosomal protein S11, 40S component
FBgn0010265	RpS13	RPS13	Ribosomal protein S13, 40S component
FBgn0011284	RpS4	RPS4X	Ribosomal protein S4, 40S component
FBgn0033902	eIF3m	EIF3M	Eukaryotic translation initiation factor 3 subunit m
FBgn0034258	eIF3c	EIF3C	Eukaryotic translation initiation factor 3 subunit c
FBgn0038834	RpS30	FAU	Fusion protein, of ubiquitin-like protein fubi and ribosomal protein S30
FBgn0002622	RpS3	RPS3	Ribosomal protein S3, 40S component
FBgn0022023	eIF3h	EIF3H	Eukaryotic translation initiation factor 3 subunit h
FBgn0002590	RpS5a	RPS5	Ribosomal protein S5a, 40S component
FBgn0034743	RpS16	RPS16	Ribosomal protein S16, 40S component
FBgn0261549	rdgA	DGKZ	Diacylglycerol kinase, mediates conversion of diacylglycerol to phosphatidic acid
FBgn0014020	Rho1	RHOC	Ras homolog gene family member C, GTPase involved in regulation of actin cytoskeleton.
FBgn0259680	Pkcδ	PRKCD	Protein kinase C delta
FBgn0283472	S6k	RPS6KB1	Ribosomal protein S6-p70-serine/threonine-protein kinase, acts downstream of mTOR signaling
FBgn0283666	Rap2l	RAP2B	Ras-associated protein 2-like
FBgn0004177	mts	PPP2CA	Catalytic subunit of protein phosphatase 2A
FBgn0038603	PKD	PRKD3	Protein kinase D, Calcium/calmodulin-dependent Ser/Thr kinase that regulates actin-dynamics
FBgn0038744	CG4733	PPP2R3B	B"/PR72 subunit of protein phosphatase 2A (PP2A) regulatory B unit
FBgn0003093	Pkc98E	PRKCE	Protein kinase C epsilon, Calcium-activated and involved in cytoskeleton control
FBgn0014011	Rac2	Rac1	Member of Rho family of GTPases
FBgn0029840	raptor	RPTOR	Raptor, component of target of rapamycin (TOR) complex 1
FBgn0003205	Ras85D	KRAS	Ras oncogene at 85D
FBgn0262866	S6kII	RPS6KA3	Ribosomal protein S6 kinase II, downstream effector and regulator of the MAP kinase pathway
FBgn0038588	CG7156	RPS6KC1	Predicted homology with ribosomal protein S6 kinase C1
FBgn0261854	aPKC	PRKCI	Atypical protein kinase C, member of the conserved Par complex
FBgn0020412	JIL-1	RPS6KA5	JIL-1 kinase involved in epigenetic gene regulation
FBgn0033935	Sin1	MAPKAP1	Stress-activated map kinase-interacting protein 1, involved in phosphorylation of Akt1
FBgn0015278	Pi3K68D	PIK3C2A	Phosphatidylinositol-4-Phosphate 3-Kinase Catalytic Subunit Type 2 alpha
FBgn0020386	Pdk1	PDPK1	Phosphoinositide-dependent kinase 1
FBgn0003091	Pkc53E	PRKCA	Protein kinase C, brain isozyme, activated by diacylglycerol
FBgn0033653	CG13192	GNB1L	Predicted homology with G protein subunit beta 1
Fbgn0033555	RpS15Ab	RPS15A	Ribosomal protein S15Ab, 40S component
FBgn0010333	Rac1	RAC1	Member of Rho family of GTPases
FBgn0027515	CG7115	PPM1L	Mg^{2+} / Mn^{2+} protein-serine/threonine phosphatase*
FBgn0037573	eIF4AIII	EIF4A3	Eukaryotic translation initiation factor eIF4A3
FBgn0003206	Ras64B	RRAS2	Ras oncogene at 64B
FBgn0260972	alc	PRKAB1	AMP-activated protein kinase (AMPK) beta subunit glycogen binding domain
FBgn0015218	eIF4E1	EIF4E	Eukaryotic translation initiation factor 4E1, binds 7-methyl-guanosine cap structure of mRNA
FBgn0023169	AMPKα	PRKAA2	AMP-activated protein kinase α subunit, phosphorylates and inhibits acetyl-CoA carboxylase
